# Probiotics for preterm infants – time to end all controversies

**DOI:** 10.1111/1751-7915.13357

**Published:** 2019-01-13

**Authors:** Gayatri Athalye‐Jape, Sanjay Patole

**Affiliations:** ^1^ Neonatal Directorate King Edward Memorial Hospital for Women Perth WA Australia; ^2^ Centre for Neonatal Research and Education University of Western Australia Perth WA Australia

## Abstract

Mortality, necrotising enterocolitis (NEC), late onset sepsis (LOS) and feeding intolerance are significant issues for very preterm (< 32 weeks) and extremely preterm (< 28 weeks) infants. The complications of ≥ Stage II NEC [e.g. Resection of the gangrenous gut, survival with intestinal failure, recurrent infections, prolonged hospital stay, and long‐term neurodevelopmental impairment (NDI)] impose a significant health burden. LOS also carries significant burden including long‐term NDI due to adverse effects of inflammation on the preterm brain during the critical phase of development. Frequent stopping of feeds due to feeding intolerance is a significant iatrogenic contributor to postnatal growth failure in extremely preterm infants. Over 25 systematic reviews and meta‐analyses of RCTs (~12 000 participants) have reported that probiotics significantly reduce the risk of all‐cause mortality, NEC ≥ Stage II, LOS and feeding intolerance in preterm infants. Systematic reviews and meta‐analysis of non‐RCTs have also shown that the benefits after adopting probiotics as a standard prophylaxis for preterm infants are similar to those reported in RCTs. No intervention comes close to probiotics when it comes to significant reduction in death, NEC, LOS and feeding intolerance at a cost of less than a dollar a day irrespective of the setting and baseline incidence of NEC. The common controversies that are preventing the rapid uptake of probiotics for preterm infants are addressed in this paper.

Mortality, necrotising enterocolitis (NEC), late onset sepsis (LOS) and feeding intolerance are significant issues for very (< 32 weeks), especially extremely preterm (< 28 weeks) infants. The health burden associated with ≥ Stage II NEC in preterm infants is significant (Neu, 2018). The overall NEC related mortality (~20–30%) rises to 40–45% in those with significant full thickness gut necrosis. Resection of the gangrenous gut often results in intestinal failure needing prolonged hospitalization, dependence on parenteral nutrition and central venous access, and recurrent infections. Apart from the significant economic burden, increased risk of long‐term neurodevelopmental impairment (NDI) is a serious concern; especially in survivors of surgical NEC (Neu, 2018). A policy of ‘zero tolerance to NEC’ is hence recommended (Swanson, 2013). Similar to NEC, LOS carries significant burden including long‐term NDI due to adverse effects of inflammation on the preterm brain during the critical phase of development (Strunk *et al*., 2014). Last but not the least; frequent stopping of feeds due to feeding intolerance, a poorly defined entity, is a significant iatrogenic contributor to postnatal growth failure in extremely preterm infants (Flidel‐Rimon *et al*., 2006). This is because the signs of feeding intolerance (abdominal distension, large and/or bile‐blood stained gastric residuals) cannot be differentiated reliably from those of NEC – the much feared potentially life threatening condition in preterm infants.

Prevention of prematurity, the single most important risk factor for NEC, LOS and feeding intolerance, has proven to be a difficult task. Till recently, antenatal glucocorticoids, early preferential use of breast milk, standardized feeding protocols, and strategies for prevention and treatment of LOS were the only options for reducing the risk of mortality, NEC, LOS and postnatal growth failure in preterm infants. Probiotic supplementation has recently become an attractive additional option in this field.

Over 25 systematic reviews and meta‐analyses of RCTs (~12 000 participants) have reported that probiotics significantly reduce the risk of all‐cause mortality, NEC ≥ Stage II, LOS, and feeding intolerance in preterm infants (Table 1). The validity of the results of these meta‐analyses is supported by the rigorous methodology, extremely small p values, narrow confidence intervals (CI), and no statistical heterogeneity for important outcomes such as NEC (Athalye‐Jape *et al*., 2014; Sawh *et al*., 2016; Thomas *et al*., 2017).

**Table 1:** Evidence supporting benefits of probiotics in preterm infants.^a^

	All‐cause mortality	NEC	LOS	TFF
Systematic review of RCTs: Sawh *et al*. (2016)	0.79 (0.68–0.93); *P *=* *0.003	0.53 (0.42–0.66); *P *<* *0.00001	0.88 (0.77–1.00); *P *=* *0.05	−1.2 (−2.2, −0.1); *P *<* *0.05
Systematic review of RCTs: Athalye‐Jape *et al*. (2014)				−1.5 (−2.75, −0.32); *P *<* *0.00001
Systematic review of RCTs: Rao *et al*. (2016)			0.86 (0.78, 0.94); *P *=* *0.0007	
Systematic review of RCTs: Dermyshi *et al*. (2017)	0.77 (0.65–0.92); *P *=* *0.003)	0.57 (0.47–0.7); *P *<* *0.00001	0.88 (0.69–0.96); *P *=* *0.05	
Systematic review of non‐RCTs: Dermyshi *et al*. (2017)	0.71 (0.62–0.81); *P *<* *0.00001	0.51 (0.37–0.7); *P *<* *0.0001	0.81 (0.69–0.96); *P *=* *0.01	
Systematic review of Non‐RCTs: Olsen *et al*. (2016)	0.72 (0.61–0.85); *P *<* *0.0001	0.55 (0.39–0.78); *P *<* *0.0006	0.86 (0.71–1.00); *P *=* *0.05	
Systematic review of RCTs (LMIC): Deshpande *et al*. (2017)	0.73 (0.59–0.90); *P *=* *0.003	0.46 (0.34–0.61); *P *<* *0.00001	0.80 (0.71–0.91); *P *=* *0.0009	
Systematic review of RCTs in animal models: Athalye‐Jape *et al*. (2018)^b^		0.51 (0.42–0.62); *P *<* *0.0001		

LMIC, Low and middle income countries; LOS, Late onset sepsis; NEC: Necrotising enterocolitis; RCT: Randomized controlled trials; TFF, Time to full feeds.

**a**. Data expressed as Relative risk/Odds ratios (95% Confidence interval), Mean difference (95% Confidence interval).

**b**. RCTs in animal models of NEC.

The benefits of an intervention in a RCT usually do not translate to the same extent in clinical practice for various reasons. It is therefore important to note that results of systematic reviews and meta‐analysis of non‐RCTs reporting benefits after adopting probiotics as a standard prophylaxis for preterm infants are similar to those reported in RCTs (Olsen *et al*., 2016). Furthermore, results of a comprehensive systematic review and meta‐analysis of RCTs in animal models of NEC support those from clinical RCTs and non‐RCTs (Athalye‐Jape *et al*., 2018). No intervention comes close to probiotics when it comes to significant reduction in death, NEC, LOS and feeding intolerance at a cost of less than a dollar a day irrespective of the setting and baseline incidence of NEC (Jacobs *et al*., 2013; Ofek Shlomai *et al*., 2014; Rao *et al*., 2016; Deshpande *et al*., 2017). Therefore it is not surprising that probiotics are considered as the miracle cure of this century in neonatology (Dermyshi *et al*., 2017). The question why probiotics have not been adopted universally is hence important.

We review the evidence behind common controversies that are preventing the rapid uptake of probiotics for preterm infants. Our results are expected to guide research and clinical practice in the field.

1. *Strain specificity:* Meta‐analysis of data from studies with different probiotic strains and protocols is often considered inappropriate given the broad consensus that probiotic effects are strain‐specific (Barclay *et al*., 2007). This approach overlooks the fact that the question addressed by systematic reviews was ‘Are probiotics *in general*, beneficial for preterm infants?’ The consistently observed benefits in various trials supported that as a class of intervention, probiotics were beneficial for preterm infants. Ganguli and Walker (2011) commented that although data demonstrate strain‐specific immunologic effects, a consistently decreased risk of NEC in trials using variable probiotic regimens suggested strain nonspecific protection (Ganguli and Walker, 2011). Vandenplas and Veereman‐Wauters (2012) agreed that the consensus about strain specificity is important but clinical data supporting this concept is limited (Vandenplas and Veereman‐Wauters, 2012). Sanders *et al*. (2018) recently reported on shared mechanisms among probiotic taxa to explain the ‘general probiotic claims’ (Sanders *et al*., 2018). They provide crucial scientific evidence on shared mechanisms of common probiotic strains that are sub‐species‐specific, species‐specific or genus‐specific. They point out that ‘a strain that has not been tested in human efficacy trials may meet the minimum definition of the term “probiotic” if it is a member of a well‐studied probiotic species’ (Sanders *et al*., 2018). It is clear that pooling of data on commonly used genus, species or sub‐species of probiotic is justified. The results of previous meta‐analyses are hence valid. Strain‐specific systematic reviews are equally important to guide research and clinical practice.
2. *The PIPs trial (UK) results:* The negative results of this large multicentre (*n *= 1310, Median gestation: 28 weeks) RCT added to the controversies about probiotics for preterm infants (Costeloe *et al*., 2016). Compared with placebo, *Bifidobacterium breve* BBG‐001 had no significant benefit on any of the primary outcomes. The need for testing every strain separately in adequately powered RCTs was emphasized and the validity of previous meta‐analyses was challenged based on strain specificity of probiotics (Costeloe *et al*., 2016). The possible reasons for the results of PIPs trial include low dose, significant cross‐contamination, random variation, and an ineffective strain (Deshpande *et al*., 2016). However, irrespective of the arguments, it is important to appreciate that the wide uncertainty (confidence) intervals for all outcomes mean significant benefit or harm of probiotic supplementation could not be ruled out [e.g. NEC: Adjusted RR: 0.93 (95% CI: 0.68–1.27); LOS: Adjusted RR: 0.97 (95% CI: 0.73–1.29]. To put it simply, the results of this trial are ‘inconclusive’ and not negative. Furthermore, putting complicated statistics aside, significant benefits for all outcomes were noted in infants ‘colonised’ with the probiotic (Deshpande *et al*., 2016).
3. *Inadequate data on extremely preterm infants:* Considering that RCT data is available from ˜2000 extremely preterm infants, and Denkel *et al*. (2016) have reported data on 4600 extremely preterm infants, quoting inadequate data as the reason for not using probiotics in this (most deserving) population is incorrect. In fact the benefits of routine probiotic supplementation were as dramatic as those in very preterm infants in the report by Denkel *et al*. (2016).
4. *Probiotics in breastmilk vs. formula‐fed infants:* Many believe that probiotics are not required if the infant is fed breast milk – the ideal food provided by nature that contains many bioactive elements including probiotics, human milk oligosaccharides, and lactoferrin. The results of two non‐RCTs are important in this context (Repa *et al*., 2015; Samuels *et al*., 2016). Repa *et al*. (2015) reported overall no significant impact of probiotics on NEC. However, NEC was significantly reduced in probiotic group infants fed any breastmilk [20/179 (11.2%) vs. 10/183 (5.5%); *P* = 0.027]. No benefits were noted in exclusively formula‐fed infants [4/54 (7.4%) vs. 6/44 (13.6%); *P* = 0.345] (Repa *et al*., 2015). Samuels *et al*. (2016) reported that introduction of probiotics was associated with reduced adjusted odds for ‘NEC or sepsis or death’ only in exclusively breastmilk‐fed infants [OR: 0.43, 95% CI: 0.21–0.93, *P* = 0.03]. Our non‐RCT supports the benefits of probiotics in breastmilk‐fed preterm infants (Patole *et al*., 2016). The reasons why probiotics may not benefit formula‐fed infants to the same extent as those fed breastmilk are easy to understand; no formula could ever replicate breastmilk with its many bioactive components.
5. *Long‐term adverse effects:* The results of a recent systematic review and meta‐analysis of studies assessing long‐term neurodevelopment of preterm infants enrolled in probiotic RCTs (*n* = 7) are reassuring in this context (Upadhyay *et al*., 2018). Six of the 7 RCTs enrolled preterm infants < 33 weeks. Outcomes were assessed at ≥18–22 months of corrected age in 5/7 RCTs. Probiotics had no effect on cognitive and motor impairment, cerebral palsy, visual, and hearing impairment (Upadhyay *et al*., 2018). Probiotics are potentially neuroprotective given their anti‐inflammatory properties, and ability to reduce NEC, LOS, feeding intolerance, and modulate the gut‐microbiota‐brain axis. Further long‐term data are important to assess this potential benefit of probiotics.
6. *Probiotic sepsis:* The reports of probiotic sepsis and the death of one preterm infant due to fungal sepsis from a contaminated probiotic product justify the concern about probiotic supplementation in preterm infants (Centers for Disease Control and Prevention, 2014; Bertelli *et al*., 2015; Esaiassen *et al*., 2016). However, it is important to know that probiotic sepsis is easy to diagnose and treat compared to the serious hospital acquired infections they prevent. The cost‐benefit ratio is very much in favour of probiotics considering the data from over 12 000 preterm infants who have received probiotics in RCTs and non‐RCTs. Independent product quality checks, and onsite laboratory back up is important to optimize safety of probiotics (Deshpande *et al*., 2011).
7. *Probiotic availability:* Limited/no access to high quality probiotics is often quoted as a reason for not using probiotics. If supporting data from well‐designed RCTs, cluster RCTs, non‐RCTs and studies assessing long‐term neurodevelopmental outcomes are available, we see no reason, why such strains/products cannot be accessed (Chou *et al*., 2010; Janvier *et al*., 2014; Totsu *et al*., 2018). Importing lifesaving drugs should not be difficult in the 21st century.

## Conclusions

In summary, there is no convincing evidence to support the ongoing controversies about probiotics for preterm infants. The devil has always been in the details but faced with the mountain of evidence, and our accountability towards parents, it is time to look at the big picture. Most of the gaps in knowledge (optimal strain/s/combinations/dose etc.) could be addressed by continued research while providing probiotics as a standard prophylaxis for preterm infants (Aceti *et al*., 2018).

## Conflicts of interest

None declared.
